# Forensic DNA Profiling: Autosomal Short Tandem Repeat as a Prominent Marker in Crime Investigation

**DOI:** 10.21315/mjms2020.27.4.3

**Published:** 2020-08-19

**Authors:** Udogadi Nwawuba Stanley, Abdullahi Mohammed Khadija, Adams Tajudeen Bukola, Imose Omusi Precious, Esewi Ayevbuomwan Davidson

**Affiliations:** 1 Centre for Forensic Programmes and DNA Studies, University of Benin, Benin City, Nigeria; 2 Molecular Drug Metabolism and Toxicology, Department of Biochemistry, College of Medicine, University of Ibadan, Ibadan, Oyo State, Nigeria; 3 Defence Headquarters Garrison, Abuja, Nigeria; 4 Department of Medical Laboratory Science, College of Medicine, University of Benin, Benin City, Nigeria; 5 Department of Biochemistry, Faculty of Life Sciences, University of Benin, Benin City, Nigeria

**Keywords:** forensic DNA profiling, short tandem repeat typing, DNA database, DNA profiling, forensic genetics

## Abstract

Short tandem repeat (STR) typing continues to be the primary workhorse in forensic DNA profiling. Therefore, the present review discusses the prominent role of STR marker in criminal justice system. All over the world, deoxyribonucleic acid (DNA) profiling provides evidence that may be used to convict criminals, as an irrefutable proof of wrongful convictions, invaluable links to the actual perpetrators of crimes, and could also deter some offenders from committing more serious offences. Clearly, DNA profiling tools have also aided forensic scientists to re-evaluate old cases that were considered closed as a result of inadequate evidence. In carrying out this review, a comprehensive electronic literature search using PubMed, ScienceDirect, Google Scholar and Google Search were used, and all works meeting the subject matter were considered, including reviews, retrospective studies, observational studies and original articles. Case reports presented here, further demonstrates the crucial role of forensic DNA profiling in mitigating and providing compelling evidence for the resolution of crimes. For case report 1, there was a 100% match between the DNA recovered from the items found in the crime scene, and the suspect’s DNA sample collected via buccal swab following 15 STR loci examination. Case report 2 further highlights the indispensable contribution of DNA database in solving crime. Therefore, it has become very necessary for developing countries like Nigeria to develop a national DNA database and make policies and legislatures that will further expand and enable the practice of forensic genetics, particularly DNA profiling.

## Introduction

The word ‘forensic’ originates from *forensis*, a Latin word meaning ‘of or before the forum’. The history of the term originates from Roman times where a criminal case presented before the public in a forum was decided in favour of the individual with the best argument and delivery (1). As an adjective ‘forensic’ means ‘pertaining to, or used in courts’, hence forensic science is the application of scientific expertise for the resolution of legal disputes, both criminal and civil. Often the term ‘forensics’ is used interchangeably (short form) with forensic science; however, it has many contested interpretations (2). Forensic science covers a broad range of specialties such as, criminalistics, digital evidence analysis, fingerprint expertise, dentistry/odontology, nursing, pathology, toxicology and questioned documents. Criminalists are dedicated to the identification, individualisation and reconstruction of physical evidence using natural science, logic and critical thinking (3).

Analysis of human genetic variations has been the impetus for advancement in forensic genetics. Until 1980s, identification (determining the nature of sample) and individualisation (uniqueness) of biological evidence relied on applications of histology, microscopy, immunology, biochemistry and serology. Traditional genetic markers such as blood group antigens, red cell isozymes, serum/plasma proteins, haemoglobin variants and human leukocyte antigen (HLA) system were used for the individualisation of blood and body fluid evidence (4). Regardless of the then significant contribution of protein markers to criminal justice, there was a prominent disadvantage with their limited degree of variability (high match probability). Although with these methods a fraction of the population could be ‘excluded’ as possible source, to say that the sample ‘did come’ from a specific person deoxyribonucleic acid (DNA) typing technologies had to be awaited. The discovery of hypervariable polymorphisms in human genome, also called as variable nucleotide tandem repeats (VNTRs) or minisatellites by Alec Jeffreys paved way for DNA finger printing using multilocus probes (MLP). The match probabilities were so low that only monozygotic twins could theoretically share DNA fingerprints. The restriction fragment length polymorphism (RFLP) technique is also based on VNTRs. However, this simple pattern match which is less sensitive, technically cumbersome, requiring microgram quantities of high molecular weight DNA led to the use of single locus probes (SLP) that analyse unique minisatellite species under more stringent conditions. DNA profiling using multiple SLPs can accumulate sufficient evidence to individualise the DNA of the sample in question (5). The issues of quality and quantity in forensic samples as well as the time required for SLP analysis were addressed by polymerase chain reaction (PCR) based methods exploring the minisatellite repeats, as in the amplified fragment length polymorphism (AMFLP) technique. Short tandem repeats (STRs) or a microsatellite consisting of short repeating motifs contained within a small fragment size are, however, more suited for PCR methodology, also shorter amplicons are feasible when there is possibility of template DNA being compromised. STR profiling, which is the focus of this review, has become the gold standard in forensic DNA profiling (6). STRs of 3 bp–4 bp repeats are highly polymorphic and profiling of 4–6 loci, or multiplexing can additionally minimise matching between unrelated individuals (7). The review discusses the prominent role of STR marker in criminal justice system and provides an excellent guide for budding forensic scientists in the area of DNA profiling, particularly in Nigeria and other developing countries where forensic studies is currently at the neophyte stage.

## Methods

This review was carried out by a comprehensive electronic literature search using PubMed, Science Direct, Google Scholar and Google Search. The following keywords and their combinations were used; forensic DNA profiling, forensic genetics, STR typing, DNA database, molecular tools in forensic DNA profiling and forensic science. All works relevant to the subject matter were considered, including reviews, meta-analyses, organisation recommendations and original articles. Preference was placed on the most recent papers but did not exclude commonly sited and highly regarded older publications.

## An Overview of DNA

Scientists wrangled about which molecule carried life’s biological instructions for many years. Most of the scientists believed that DNA was too simple a molecule to play such a critical role. Rather, they thought that proteins were more likely to carry out this vital function because of their greater complexity and wider variety of forms (8). An understanding of the critical role of DNA as the genetic material became clear by 1952 after the ground breaking experiments by Alfred Hershey and Martha Chase. In 1953, the work of James Watson, Francis Crick, Maurice Wilkins and Rosalind Franklin, on X-ray diffraction patterns, significantly contributed in figuring out the double helix structure of DNA; a structure that enables it to carry biological information from one generation to the next (8).

As a matter of fact, DNA is virtually found every cell of the human body and is predominantly located in the cell nucleus (nuclear DNA), but a fraction of DNA can also be found in the mitochondria (mitochondrial DNA or mtDNA) (9). DNA, along with the instructions it contains, is passed from parents to their offspring during reproduction (8). Similar to fingerprints, every person has a unique DNA signature that remains unchanged throughout their lives. DNA testing, generally called DNA profiling, takes advantage of the fact that, with the exception of homozygous twins, the genetic material of each person is unique and is an omnipresent residue that trails us wherever we go (10).

The human ‘genome’ has been completely read, if not translated, after a monumental sequencing effort, and today, it is known that the 3 billion bases of human genome that are distributed among 23 chromosomes, houses fewer than 30,000 genes (codes for protein), representing less than 5% of its length. The human genome project confirmed what scientists had already known, that the noncoding regions of the genome contain, among other things, tracts of repetitive sequences (11). The single-locus satellites are localised at a specific site of a given human chromosome, while multilocus satellite elements or STRs are spread throughout the entire genome (12).

## Repetitive DNA Sequences

Repetitive DNA sequences can be moderately or highly repetitive, tandemly or dispersely organised within the eukaryotic genome. Satellite DNAs, minisatellites and microsatellites constitute highly repetitive tandem sequences (VNTRs) (13). The word ‘satellite’ corresponds to the lower density (satellite) fraction of repetitive DNA fragments after density gradient centrifugation. Minisatellites have heterogeneous array of 10 bp–100 bp core repeat motifs that extends to 1 kb–15 kb, they are distinct from microsatellites in structure and function and are single locus satellites localised to specific sites in the human genome. Homogenous array of repeat motifs of 2 bps–6 bps with repeat size of less than or around 1 kb are called microsatellites or STRs (14). VNTR region has a high rate of mutation which is 10 to 100,000 times higher than the average rate at other genomic sites, variations in the number of repeat units also contributes to polymorphisms in loci. Strand-slippage during DNA replication and unequal crossing over are responsible for the expansion or contraction of repeat units. Different people in a population thus differ in repeat number and in the order of repeat types (principle of digital DNA typing); independent assortment further also causes allelic variations within an individual in autosomal repeat sites. Though minisatellite polymorphisms were explored in many forensic investigations, the abundance of STR markers along with its PCR compatibility made it a method of choice among scientists (13).

## DNA Evidence and Forensic Science

Forensic crime investigation owes its justification to the Locard’s exchange principle, ‘every contact leaves a trace’. It states that there is an exchange of material between two objects during contact leaving a trace. Although, traces found at the crime scene are often populated with multiple evidence, intermixing of the remains of victims, or severe fragmentation, which renders the traditional identification based on anthropological and physical characteristics of the victim inconclusive and inefficient (15). But, DNA profiling, a gold standard in resolving forensic cases and providing pinpointed identification of victims, and also suspects in some instances, remains a veritable tool in situations of multiple evidences (15, 16). About 99.9% sequence of DNA is reported to be the same in all humans with only about 0.1% variation, and the odds of two persons not related by blood having the exact same DNA sequence is about 1 in 594.1 trillion individuals (17). In the light of this, of course, DNA testing has exonerated the innocent and convicted the guilty (18). It has been reported that within several jurisdictions samples collected from touched objects represent more than half the total number of samples processed for DNA profiling (16). This is in part due to the finding that DNA can be detected from non-visible biological material left on a surface merely through touching it by hand, and one contact event can simultaneously include both direct/primary and indirect/secondary transfer events (16, 19). A self DNA deposited within the handprint may be regarded as a direct deposit, whereas a non-self-component is regarded as an indirect deposit (20, 21). Other prominent biological material for DNA extraction and profiling includes: saliva, blood, nails, tooth and hair strand.

DNA fingerprinting was first used in forensic science in 1986 when police in the United Kingdom requested Dr Alec J. Jeffreys, a genetics professor at the University of Leicester to profile a suspect of rape and murder of 15-year-old Dawn Ashworth in Leicestershire (22, 23). Prior to the case, Dr Jeffreys had discovered that patterns in some regions of a person’s DNA could be used to distinguish one person from another (22, 23). The suspect of the case was Richard Buckland, who had even confessed to Ashworth’s murder. Following the DNA profiling analysis of the samples from the 1983 and 1986 crime scenes and from Buckland, Dr Jeffreys found matching DNA from both crime scenes. However, the recovered DNA did not match Buckland’s DNA profile (12, 22, 23). In an attempt to find the real culprit, the police undertook a genetic dragnet; obtaining blood and saliva samples from over 4,000 men in the Leicestershire area between the ages of 17 years and 34 years, a conclusive match was not found. However, a man was overheard saying that he was paid to pose as someone else and provided false samples. The person trying to evade the DNA dragnet was Colin Pitchfork. When Pitchfork’s DNA was profiled, it matched the crime scene samples. Pitchfork was arrested on the 19th September 1987, convicted, and sentenced to life in prison the following January. He was the first murderer to be convicted using DNA analysis (22, 23). In the same year of Pitchfork sentence (1987), in the United States, Tommy Lee Andrews was convicted in a rape case based on DNA evidence, where his genetic code was matched with that of semen traces recovered from the victim (12).

Other important cases that strengthened the use of DNA evidence includes the case of Glen Dale Woodal versus the State of West Virginia in 1992 and the multiple murder trial of Timothy Wilson Spencer versus the State of Virginia in 1994 (12). The DNA evidence in the Woodal case exonerated him while that of the Spencer case resulted in his conviction and sentencing to death penalty (12). Since the case of Pitchfork in 1987, significant scientific research and resource has been devoted to the development and refinement of DNA analysis technologies. Notably, in 1995 the United Kingdom National DNA Database was established to enhance the investigative use of DNA profiles, and on a global scale, most countries now use forensic DNA analysis in one form or another (23). The main questions that a forensic DNA scientist is asked to address are as follows: whose DNA is it, from what body fluid was it originated, how did it get there, and have the results been reported in a fair and balanced way? (23).

In recent times, the admissibility of DNA evidence is widely used in many courts around the world. Its underpinning science is reliable, reproducible and accurate, and based on validated technology and techniques for both the generation of a DNA profile and the interpretation of that profile (23). In accordance with the United States National Research Council’s (NRC) 1996 report on DNA evidence ‘the state of the profiling technology and the methods for estimating frequencies and related statistics have progressed to the point where the admissibility of properly collected and analysed DNA data should not be in doubt’ (12, 24, 25).

## STR Typing

DNA fingerprinting technique began years ago with the introduction of restriction fragment length polymorphism (RFLP), and in the 1990s, RFLP method gave way to PCR methodologies that had the advantage of being able to amplify DNA (11, 26). After several improvements and refinements to the PCR-based tests, the forensic community came to an agreement on the use of STRs (11, 15, 27). Although there are other DNA markers in use, STR typing is the method of choice for most forensic laboratories, and given the investments in infrastructure, training, databases, and accreditation, it will be for the foreseeable future (11, 28). Evidentially, a wide range of microsatellite loci have been identified, characterised, and demonstrated to be highly abundant in the human genome (29, 30). The high abundance and polymorphic nature of STR loci was a vital factor considered for incorporation into commercial kits by manufacturers, and has ever since then remained the most frequently applied methodology, and the current gold standard for human identification in forensic laboratories (15, 26, 31).

Analysis involving STR typing follows a general methodology of DNA profiling. However, the methodology for STR typing relies on the standard operating procedures provided by the manufacturers of the commercial kit for use in forensic laboratory. STR typing involves the general steps for DNA profiling in the following order: isolation of DNA by a process called DNA extraction, quantification of the DNA in the sample, amplification of STR loci, separation of the PCR amplicons on a genetic analyser using bioinformatics to analyse the resulting data and comparing the data from one specimen to databases housing previously generated STR sets (32, 33). Repetitive DNA sequences with varying numbers of repeats, referred to as STR loci, are amplified using primers with differently coloured fluorophores and these amplicons are distinguished by both size and color (33). Additionally, in performing analysis on STR loci, the invariant flanking regions surrounding the repeats must be determined and once the flanking sequences are known, then PCR primers can be designed and the repeat region amplified for analysis (34). There are two usual ways to identify STR. These include either searching the DNA sequence databases such as CODIS or GenBank for regions with more than six or so vicinal repeat units or performing molecular biology isolation methods (29, 35, 36).

**Figure 2:**
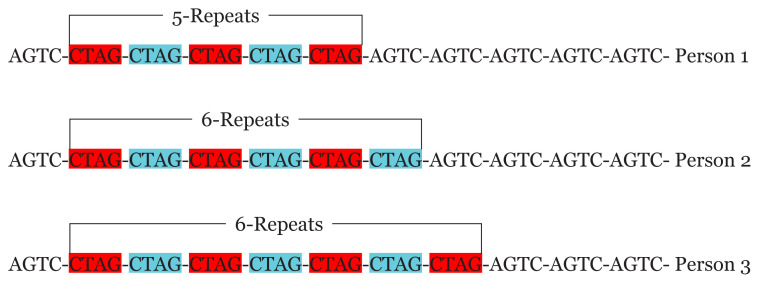
Showing tetra-nucleotide (CTAG) STRs of different lengths at the same locus

STRs are highly polymorphic and alleles of the STR loci are differentiated by the number of copies of the repeat sequence within each of the STR locus (12). Research findings have demonstrated that the more STR loci being used for typing, greater the discrimination value (12, 15, 37), since the probability that two individuals taken from a random population possessing exactly the same number of repeats units for all the STR being analysed, is extremely rare (12). They can vary in size from person to person without impacting the genetic health of the individual (34). For example, at the same locus, a tetra-nucleotide repeat sequence (represented by CTAG) will vary from one person to the other as represented in Figure 2. Person 1 has 5 repeats, person 2 has 6 repeats and person 3 has 7 repeats.

Most STRs are found in the noncoding regions, while only about 8% are located in the coding regions and their density vary slightly among chromosomes (30). For instance, in humans, chromosome 19 has the highest density of STRs (35, 38). According to International Genome Sequence Consortium in 2001, on the average, one STR occurs per 2,000 bp in the human genome and from the study of Hao and Jia-You (38), it reported that the most common STRs in humans are A-rich units: A, AC, AAAN, AAN and AG. The STR locus is represented by codes composed of alphabets and numbers, for instance D13S317, where D represents DNA, 13 means chromosome 13 on which the STR locus locates, S stands for STR and 317 is the unique identifier.

## DNA Databases

The introduction of amplification technology linked to the analysis of STRs led to the availability of sufficiently sensitive and robust systems for the formation of efficient and effective DNA databases (39). A leading advancement in forensic DNA profiling was the establishment and expansion of centralised national criminal DNA databases (40, 41). Since the comprehensive legislation was enacted in 1995 forensic scientists in the United Kingdom set up the first national DNA database that would hold both personal DNA profiles together with results obtained from crime scenes (42). Primarily, the function of a criminal DNA database is to produce hits to STR sequences between stored DNA profile of suspects, convicted offenders, victims and DNA evidences found at the crime scene as allowed by legislation of the country. It is reported that around 69 countries currently operate national forensic DNA databases; others are being expanded or established in at least 34 additional countries (40). Regardless of the immense contribution of DNA database to criminal justice system as a significant resource for criminal investigation and prosecution activities throughout the world (43), arguments that operating forensic DNA databases involves potential threats to the protection of a range of human rights, in particular liberty, autonomy, privacy, informed consent, moral integrity and the presumption of innocence do exists (44, 45). It was recommended that a responsible forensic DNA database policy is required to strike a reasonable balance between these positions, create a moral and ethical spectrum involving both professionals in the area of forensics, the law enforcement, the public, and particularly, social groups which are less involved in genetics (46, 44).

A universal DNA database confers huge benefits in efficiently and effectively solving crimes (47). According to the report of the Federal Bureau of Investigation (FBI) 2019, the National DNA Index System (NDIS) contains over 13,973,206 offender profiles, 3,721,360 arrestee profiles and 973,108 forensic profiles as of September 2019 (48). However, the report further stated that the success of DNA database (CODIS) was ultimately going to be ascertained by the number of crimes it aids in solving. Consistent with the same report, of course, CODIS did produce over 485,063 hits, assisting in more than 474,576 investigations as of September 2019 (48). In the same light, the report of Federal Bureau of Investigation on federal DNA database, as available on the 30th October 2019, further highlights the importance of DNA database in solving crime. It reports that the Federal DNA Database Unit (FDDU) serves the greater forensic community by aiding investigations through hit confirmations against individuals whose profiles are in the NDIS (49).

Additionally, forensic DNA database is a computer database containing records of DNA profiles and it constitutes an important investigative resource in contemporary criminal justice systems (50, 51). The incredible power of DNA technology as an identification tool had brought a tremendous change in criminal justice system (12) and many countries now operate forensic DNA-databases to identify owners of crime related stains (52). The centralised and computerised storage of DNA profiles in a database enables the systematic comparison and automated matching of crime scene samples and individual profiles (51). Using DNA to trace persons who are suspected of committing a crime has been a major advance in policing and when DNA profiling is used efficiently, it can help to convict people who have committed serious crimes or exonerate people who are innocent (50). DNA database remains an information resource for forensic DNA typing and STR DNA markers continues to be a prominent player (12).

After over a decade of operation, the NDIS continues to grow in importance and size, alongside STR DNA technology (53). The United Kingdom Forensic Science Service (FSS) was at the forefront in application of STR markers to forensic case work with a first-generation quadruplex (STR loci) namely; TH01, vWA, FES/FPS and F13A1 (54). A second-generation multiplex (SGM) followed a few years later and six STR loci (TH01, vWA, FGA, D8S1179, D18S51 and D21S11) and the sex-typing marker amelogenin were examined (27, 55). National DNA databases began to be developed, with the first one being the United Kingdom National DNA Database in April 1995, comprising of the SGM loci and in October 1998, the United States launched its (NDIS) containing 13 core STR loci (TH01, vWA, FGA, D8S1179, D18S51, D21S11, CSF1PO, TPOX, D3S1358, D5S818, D7S820, D13S317and D16S539) (27, 34). Clearly, thousands of polymorphic microsatellites (STRs) have been characterised in human DNA and it is reported that there may be more than a million STRs loci present depending on how they are counted (30) the STR loci used in the United States. CODIS database are scattered throughout the human genome (17).

The study of Budowle et al. (56) reported that, from the STR Project meeting held on 13th–14th November 1997, 13 core STR loci namely: CSF1PO, FGA, TH01, TPOX, vWA, D3S1358, D5S818, D7S820, D8S1179, D13S317, D16S539, D18S51, and D21S11 were selected to be the basis of the future CODIS national DNA database. Of the original 13 CODIS STR loci, the three most polymorphic markers are FGA, D18S51 and D21S11. TPOX, CSF1PO, and TH01 typically exhibits the least amount of variation between individuals (34) and when all 13 CODIS core loci are tested, the average random match probability is rarer than one in a trillion among unrelated individuals (57, 58). However, in January 2017, the number of loci for new CODIS profiles was increased to 21 including the sex marker, amelogenin (22).

Using the previous described classification scheme, the 13 CODIS core STR loci may be divided into four categories (58).

- Simple repeats consisting of one repeating sequence: TPOX, CSF1PO, D5S818, D13S317, D16S539;
- Simple repeats with non-consensus alleles: TH01, D18S51, D7S820;
- Compound repeats with non-consensus alleles: vWA, FGA, D3S1358, D8S1179; and
- Complex repeats: D21S11.

Originally building on the initial FSS work, a European Standard Set (ESS) of STR loci were selected in 1999 and many of the same STR loci used in the United States are also adopted in European forensic DNA laboratories (34, 59). The European community, working through the European Network of Forensic Science Institutes (ENFSI) and the European DNA Profiling Group have continued to improve, and developed a strategy to expand the ESS of loci and encourage standardisation (60, 61). Correspondingly, the FBI also launched a review of the current CODIS core loci to determine if additional loci should be included in the CODIS core to facilitate greater discrimination, assist missing person investigations and promote compatibility for international data sharing efforts (53). In Africa, South Africa does in fact currently have a National DNA Database for Criminal Intelligence, and is still in the early stages of recognising the importance of maximising the size of its National DNA Database in order to enhance its capacity to solve cases with DNA evidence (10).

The expense, time and effort needed to develop DNA databases as suggested by ‘DNA project. Fighting crime with science’ (10), are justified by the facts that:

- Criminals tend to re-offend. For example, 90% of rapists and 50% of armed robbers have a previous conviction.
- The severity of crimes committed by repeat-offenders often increases over time, with criminals committing their first offence between the ages of 16 and 19 years.
- A small number of criminals are often responsible for numerous crimes. DNA databases can assist in linking these crimes to one another.

## Cases Resolved with the Use of STR Typing

**Figure 4:**
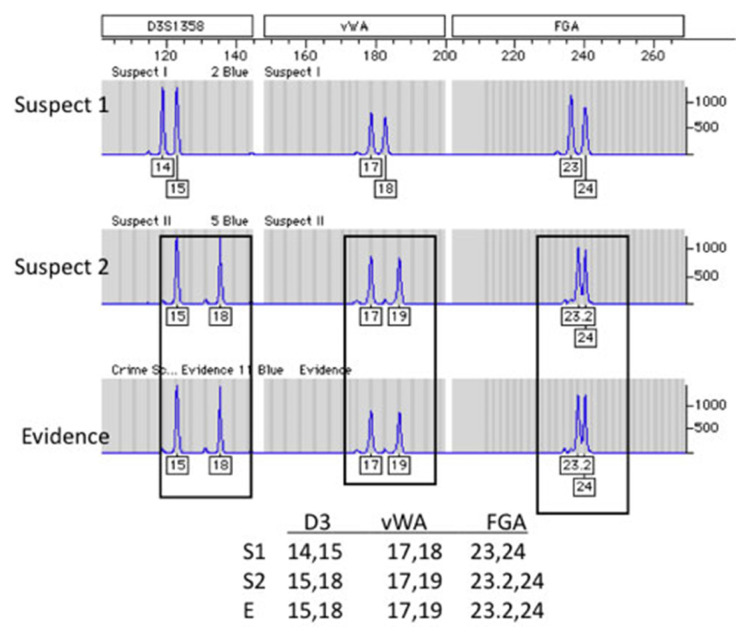
A comparison between a crime scene evidence and two suspects, adopted from (10)

At a scene of crime, blood stains, semen, other biological traces or the body of the victim are found very often and any of these could be used as evidence. The forensic scientist now uses the evidence before him to link the case to the arrested suspect through the matching of the trace evidence to the suspect, using DNA profiling (50). For cases in which no suspect can be identified, DNA samples collected at the scene of crime may be compared with DNA profiles stored on a National DNA Databases for a match or hit between the crime scene evidence and a database profile and then open an investigation on individuals with perfectly matched profiles (10, 50, 62). A simplified example is depicted in Figure 4, a comparison between a crime scene sample and two suspects. In this case, biological trace were collected from the scene of crime and represented as evidence, samples from suspects were collected as well. It is clear that the evidence taken from the crime scene matches the DNA profile of suspect 2, as the repeat sequence of the three (3) STRs Loci (D3S1358, Vwa, and FGA) adopted were identical to the evidence (10).

### Case Report 1

Case report 1 is stated as reported by Jakovski et al. (50). In a village near by the city of Kicevo a married couple was found killed and bodies were corded. During the autopsy, blood from the two victims, nail debris and pieces from the ropes were sent for DNA analysis. Extraction of DNA was made with Qiagen mini kit. PCR reaction was performed with identifier amplification kit and capillary electrophoresis was done using 310 Genetic analyser. DNA from the rope, which was used to tie the male victim legs matched with the autosomal STR profile from an unknown male. The presumed unknown DNA profile was sent to the Macedonian forensic DNA database stored in the forensic department of Ministry of Internal Affairs (MIA). The result was negative. After five years, there was a church burglary. During the crime scene investigation, blood was found on the broken window. The suspected person was arrested and buccal swab was taken for DNA analysis which was compared with the blood found on the broken window of the church and was a positive match. His profile was also run in the Macedonian National DNA database and there was a positive match with the DNA profile extracted of the exhibit of the double murder of the married couple. This case was, thus, solved (50). The comparison of the evidence and suspect DNA profile is represented on Table 1.

### Case Report 2

A 35-year-old man was shot dead in his backyard in an urban neighbourhood. Following a report of gunfire, police officers responded. Arrests were made, and witnesses reported that they had seen several males running from the crime scene. A thorough search was conducted at the crime scene and the surrounding area. Multiple items of evidences were found, including; a handgun, a pair of work gloves, and two shirt sleeves. An autopsy was conducted and it revealed an intermediate range penetrating gunshot wound of the head; a large-caliber bullet was recovered from within the brain, which was determined to have been fired from the handgun discovered in the alley. DNA was extracted from the work gloves and shirt sleeves using organic extraction. Quantification was conducted using an applied biosystems (ABI) quantifiler human kit with an ABI 7500 real-time PCR system. Amplification was performed using a Promega PowerPlex 16 kit (32 cycles) on a GeneAmp PCR system 9700 and subsequent electrophoresis with an ABI 3130xl genetic analyser using 3 kV eight-second injections. The result showed an autosomal STR profile from unknown male. The presumed unknown DNA profile was subsequently matched to a stored DNA profile in CODIS, a hit was found and the case was solved. The alleged shooter was charged with felony murder and attempted robbery. He was sentenced to 65 years in prison (63).

## Discussion

All over the world, DNA profiling provides evidence that may be used to convict criminals, irrefutable proof of wrongful convictions, invaluable links to the actual perpetrators of crimes and could also deter some offenders from committing more serious offenses (10, 12). Clearly, DNA profiling tools have also aided forensic scientists to re-evaluate old cases that were considered closed as a result of inadequate evidence. The innocent project is one of such prominent insights where DNA profiling tool aided in the exoneration of wrongfully convicted people. The United States-based organisation was founded in 1992 and to date has been instrumental in successfully exonerating 272 people, 17 of whom served time on death row.

In the present study, case reports 1 and 2 also demonstrate the crucial position of forensic DNA profiling in mitigating and providing compelling evidence for the resolution of crimes. In both case reports, the suspects would not have been found guilty of the crime but for the intervention of DNA profiling. For case report 1, there was a 100% match between the DNA recovered from the different materials found in the scene of crime (rope and gloves) and the suspect’s DNA sample collected via buccal swap following the 15 STR loci examinations. Case report 2 further emphasises the indispensable contribution of DNA database in solving crime. In this case, the crime is a reoccurring decimal for the suspect and his DNA was previously collected, profiled and stored in the DNA database. An indirect comparison was conducted and a hit was found between the profiled DNA from the work gloves and shirt sleeves recovered from the crime scene and DNA database, in this case, CODIS database.

These stated instances demonstrate that, DNA profiling and database are critical tools in solving crime, regardless of how long it was perpetrated and the continued use of forensic DNA evidence will lead to long-term savings for the criminal justice system. Therefore, it has become very necessary for developing countries like Nigeria to develop a national DNA database and make policies and legislatures that will further expand and enable the practice of forensic genetics, particularly DNA profiling.

## Conclusion

Owing to the prominent position of STRs in DNA profiling, the current review provides an explicit explanation of the use of STR in crime investigation with reference to critical case reports and historic events. We consider this review to also be a learning guide for budding forensic scientists in the area of DNA profiling, particularly in Nigeria and other developing countries where forensic studies is currently at the neophyte stage.

## Figures and Tables

**Figure 1:**
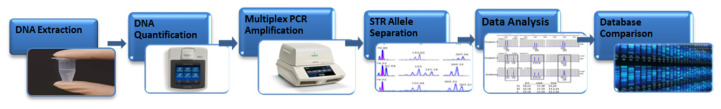
Work flow of STR typing

**Figure 3:**
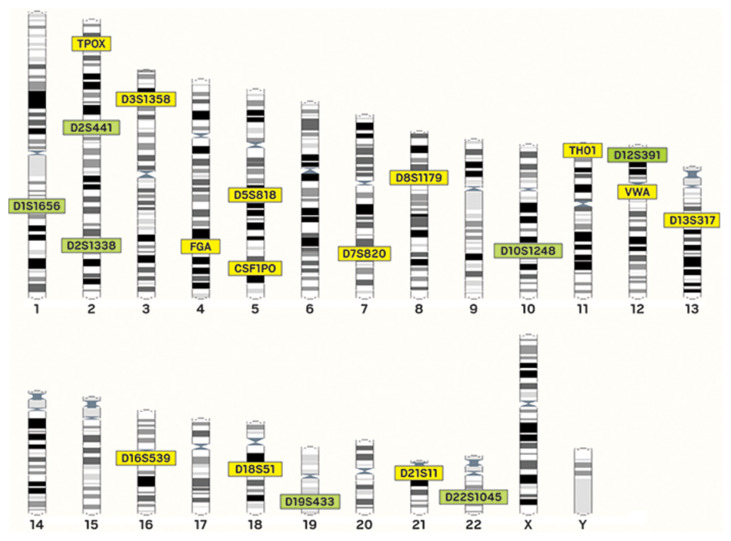
The original 13 STRs highlighted in yellow and seven added in January 2017 highlighted in green adopted (22)

**Table 1:** Profile analysis results for autosomal STR’s from the married couple victim, suspected and exhibit material

Locus	Repeat motif	Victim 1	Victim 2	Rope	Gloves	Suspected
D8S1179	Compound TCTA/TCTG	11, 15	14, 16	9, 14	9, 14	9, 14
D21S11	Complex TCTA/TCTG	28, 30	28, 30	28, 29	28, 29	28, 29
D7S820	Simple GATA	10, 12	10, 12	8, 9	8, 9	8, 9
CSF1PO	Simple AGAT	11, 12	12, 12	10, 12	10, 12	10, 12
D3S1358	Compound TCTA/TCTG	15, 16	14, 17	15, 18	15, 18	15, 18
TH01	Simple TCAT	6, 9.3	8, 9.3	6,8	6,8	6,8
D13S317	Simple TATC	11, 11	11, 13	10, 12	10, 12	10, 12
D16S539	Simple GATA	11, 11	11, 12	12, 12	12, 12	12, 12
D2S1338	Compound TGCC/TTCC	17, 17	17, 25	17, 23	17, 23	17, 23
D19S433	Simple AGAA	13, 14	12, 13	14, 14	14, 14	14, 14
vWA	Compound TCTA/TCTG	17, 18	14, 16	17, 18	17, 18	17, 18
TPOX	Simple AATG	8, 9	11, 11	11, 11	11, 11	11, 11
D18S51	Simple AGAA	13, 15	15, 17	15, 16	15, 16	15, 16
D5S818	Simple AGAT	11, 12	12, 12	12, 13	12, 13	12, 13
FGA	Compound CTTT/TTCC	21, 26	21, 23	23, 24	23, 24	23, 24
Amelogenin		XY	XX	XY	XY	XY

Note: Adopted from (34, 50)
